# Reply to Boucher, B.J. Comment on “Lopes et al. Adiposity Metabolic Consequences for Adolescent Bone Health. *Nutrients* 2022, *14*, 3260”

**DOI:** 10.3390/nu15234953

**Published:** 2023-11-29

**Authors:** Kátia Gianlupi Lopes, Elisana Lima Rodrigues, Mariana Rodrigues da Silva Lopes, Valter Aragão do Nascimento, Arnildo Pott, Rita de Cássia Avellaneda Guimarães, Giovana Eliza Pegolo, Karine de Cássia Freitas

**Affiliations:** 1 Post-Graduate Program in Health and Development in the Mid-West Region, Federal University of Mato Grosso do Sul, Campo Grande 79070-900, Brazil; kgianlupi@gmail.com (K.G.L.); elisana.lima10@gmail.com (E.L.R.); marianalopes316@gmail.com (M.R.d.S.L.); aragao60@hotmail.com (V.A.d.N.); rita.guimaraes@ufms.br (R.d.C.A.G.); 2 Institute of Biosciences, Federal University of Mato Grosso do Sul-UFMS, Campo Grande 79079-900, Brazil; arnildo.pott@gmail.com; 3 Faculty of Pharmaceutical Sciences, Food and Nutrition, Federal University of Mato Grosso do Sul, Campo Grande 79070-900, Brazil; giovana.pegolo@ufms.br

We appreciate your careful reading and comments [1] regarding our article: Adiposity Metabolic Consequences for Adolescent Bone Health [2]. The information reported by Boucher in his comment is relevant and adds to the various approaches made in the published article, expanding the possibilities for discussion on the subject.

This article presented a literature review regarding the concern about the unsatisfactory acquisition of bone mass in childhood and adolescence, which has consequences in adult life and increases the risk of developing bone diseases at a more advanced age. There is more consensual information that nutrient deficiencies, especially calcium and vitamin D, associated with a sedentary lifestyle and lack of sun exposure contribute to the unsatisfactory acquisition of bone mass, now added to recent studies that point to childhood obesity impacting impaired bone health. Obesity in childhood and adolescence has been increasing in recent years and this observation has certainly become the subject of studies to better understand the process.

Therefore, we analyzed factors related to bone health and their association with obesity and metabolic syndrome in adolescents. Among these factors, we discussed Nutritional Aspects for Growth Optimization, particularly calcium (whose intake among adolescents is generally low) and other minerals and elements which are involved in bone growth, such as bone mass constituents (magnesium and fluorine), and components of the enzymatic system involved in matrix mechanisms, such as zinc, copper, and manganese, in addition to Vitamins D, C, and K, which also play essential roles in calcium metabolism by acting as cofactors of key enzymes in bone metabolism [3]. Vitamin D deficiency can have severe clinical consequences such as hypocalcemia.

We reinforce the importance of (PUFAs), especially omega-3 fatty acids, as they have positive effects on bone mass and quality, which are possibly attributable to the reduced production of prostaglandins E2 (PGE 2) and inhibition of the receptor of the differentiated osteoclasts caused by the ligand NF-κβ [4,5].

The relationship between obesity and bone health also permeates the vital role that adipose tissue has in bone metabolism, mainly due to the production of adipokines, some of which act positively in bone formation [6]. However, obesity can also increase bone reabsorption through the increased release of pro-inflammatory cytokines, such as tumor necrosis factor-α (TNF-α) and interleukin 6 (IL-6), which stimulate the formation and activity of osteoclasts through the receptor activator of the nuclear factor kappa-beta ligand (RANKL)/(RANK)/Osteoprotegerin (OPG) pathway [7,8,9].

Furthermore, children with excess weight tend to have a stature below their potential according to the mean parental height [10]. Klein, Newfield, and Hassink [11] and Oh et al. [12] obtained similar results once both studies found an association between advanced bone age and child obesity and, although obesity is still not considered a direct cause of osteoporosis, there are signs of that correlation. Excess body weight can accelerate sexual maturity, which is reflected in accelerated bone age, mainly in obese children and adolescents.

Another point highlighted by Boucher refers to the importance of maintaining adequate levels of vitamin D during pregnancy, reducing the risk of having babies with a vitamin deficiency. This is due to the fact that women and infants are also at a high risk for vitamin D deficiency, as 73% of the women and 80% of their infants were found to have vitamin D deficiency at the time of birth, despite taking a daily prenatal multivitamins containing 400 IU of vitamin D [13]. Furthermore, there is evidence that maternal-fetal vitamin D deficiency is involved in “fetal programming” or epigenetic modifications, which may cause damage to bone mineralization and changes in the body composition of their offspring [14].

It is possible to understand that there are several critical points to be observed and studied in the relationship between childhood obesity and damage to bone health. In the midst of so much information, new original studies, especially with tracking possible risk factors, as well as reviews, are welcome, as these are fundamental for the selection of variables that highlight new investigations in population studies, given the complexity of the subject. The information cited by Boucher confirms other aspects that must be carefully analyzed and added together in the search to prevent bone damage that can begin in the early stages of life and extend throughout life. Furthermore, we are grateful for the collaboration, in order to provide the group with other reflections on the topic, in order to expand future studies. This context includes monitoring the vitamin D levels in pregnant women and repercussions during childhood and adolescence, also considering the diversity in the level of prenatal care and sociodemographic and economic factors involved. Finally, the comments strengthen our concern about the topic, especially for population groups at greater risk.
